# Adherence to Oral Contraception in Young Women: Beliefs, Locus of Control, and Psychological Reactance

**DOI:** 10.3390/ijerph182111308

**Published:** 2021-10-28

**Authors:** Ascensión Fumero, Rosario J. Marrero, Wenceslao Peñate, Juan M. Bethencourt, Pedro Barreiro

**Affiliations:** 1 Departmento de Psicología Clínica, Psicobiología y Metodología, Facultad de Psicología, Campus de Guajara, Universidad de La Laguna, 38200 San Cristóbal de Tenerife, Spain; rmarrero@ull.edu.es (R.J.M.); wpenate@ull.edu.es (W.P.); jmbethen@ull.es (J.M.B.); 2 Servicio Canario de la Salud, 38071 San Cristóbal de Tenerife, Spain; rinverbarin@gmail.com; 3 Gabinete Mente y Salud, 38003 San Cristóbal de Tenerife, Spain

**Keywords:** hormonal oral, medication, adherent patient, internal–external control, reacting, beliefs concerning, attitudes

## Abstract

Background: There is a high dropout rate of oral contraceptive pills (OCP), mainly due to a lack of adherence to treatment. The aim of this study was to identify the psychological processes and attitudes toward medication involved in adherence to OCP, depending on the prescription, to avoid unintended pregnancies (AUP) or gynecological problems (GP). Methods: This cross-sectional study was conducted by asking 689 young women in the fertile period, mean age 23.41 (SD = 5.90), to complete questionnaires related to attitudes, beliefs, psychological reactance, locus of control, and adherence to contraceptive medication. Descriptive analyses and a binary logistic regression were performed. Results: The results confirmed that different beliefs and psychological processes were involved in adherence to oral contraception, based on women’s reasons for taking contraceptive medication. More psychological processes were involved in non-adherence in the AUP group than in the GP group. Psychological reactance contributed most to explaining non-adherence in women who used the OCP to prevent unintended pregnancies. Conversely, women with gynecological problems reported difficulties in adherence, mainly due to their beliefs about contraceptive pills. Conclusions: These findings indicate that attitudes toward medication and psychological processes can play an important role in adherence to OCP, including reasons for using the pill. Identifying the psychological factors and beliefs linked with contraception could guide health professionals to provide counseling to women, thus increasing their adherence to medication and maximizing their health and well-being.

## 1. Introduction

Oral contraceptive pills (OCP) are a medical milestone associated with two main health benefits. On the one hand, OCP are prescribed among healthy women of childbearing age to avoid an unintended pregnancy (AUP). On the other, OCP allows the treatment of headaches and migraines [1], joint or inflammatory pain [2], irregular menstruation, and acne associated with gynecological pathologies (GP) [3]. Although most women use OCP for contraceptive purposes, approximately 14% do so for non-contraceptive reasons [3]. Similar adherence rates have been found for gynecological and contraceptive treatments [4], with a high dropout rate (almost 50%) of OCP within the initial 12 months of beginning the treatment, mainly due to the onset of side effects, and a lack of adherence to treatment [5,6]. Non-compliance with contraceptives methods is a serious problem. In 2019, Spain registered 99,149 cases of voluntary interruption of pregnancy: a rate of 11.53 per 1000 women [7]. This failure to comply with guidelines of contraceptive medication is one of the reasons leading to high economic and clinical costs [8].

In cases of unintended pregnancy, in addition to the probability of pregnancy, the consequences of dropping out of OCP entail a higher rate of abortions [9], the risk of poor care of the newborn in women with limited resources [10], perinatal maternal depressive symptoms [11], and difficulties in family planning [12]. Moreover, in cases of gynecological problems, failure to continue OCP could lead to difficulties in controlling symptoms, exacerbation of disease, and greater distress. Thus, the prevalence of dysmenorrhea is more likely in women not using OCP [13], though in polycystic ovary syndrome, the use of OCP can have adverse consequences [14]. In fact, the main reason for non-adherence is the fear of becoming seriously ill [15], while prior use of such treatment and educational talks about the problems of hormone therapy significantly improve the continuation rate, preventing the various problems for which it was prescribed. Comparing both groups of women who use the pill for different health reasons would allow us to know if the voluntary choice of the pill in AUP is associated with greater adherence and psychological processes linked to self-control and emotional factors, while in cases of GP adherence it would be linked to beliefs about medication. The literature addressing adherence to hormone medication indicates that behavioral factors are not the only robust predictors of adherence [16]. Attitudes toward medication and the psychological processes involved can play an important role in adherence to OCP. Similarly, the reasons why hormonal treatment is used may also modulate adherence to the medication.

The patient-related factors, such as forgetfulness, false information, misconceptions, irrational beliefs, and a lack of skills, have been isolated as factors affecting adherence to OCP [17]. Young women often fail to use contraceptives correctly and/or consistently. Approximately 39% forgot to take the contraceptive pill at least once in the past month or did not take it at the same time each day. The majority attribute this misuse to stress and preoccupations [18]. Not having a correct pill-taking routine, the resting periods of the pill, a failure to read and understand the written information of the OCP, and the occurrence of side effects, are among the factors associated with poor adherence, missing pills, or an early discontinuation of OCP [19].

The decision to adhere or not to medication is often a voluntary act that, together with its multifactorial nature, has led to a shift in research focus toward the psychological characteristics involved [20]. Studies highlight the importance of socio-cognitive factors, such as self-regulation, self-efficacy, necessity and concerns of medication beliefs, perceived barriers, and perceived susceptibility [21,22]. In this sense, external–internal control processes could be relevant in the decision to take a certain medication [23,24]. A health-related locus of control refers to a person’s belief about the causes and consequences of their health. The internal locus of control has been found to predict adherence to treatment [25,26]. Additionally, women who are internally motivated to take medication show better adherence than those motivated by external factors [27]. Shared decision-making about contraceptive use between women and health care providers improves adherence, although many women prefer to base their decision on their personal experience and make decisions autonomously [28].

Similarly, emotional factors linked to the perception of risks or benefits generated by the medication, which could be assessed by psychological reactance, could explain the phenomenon of adherence or non-adherence to medication. Emotional and cognitive factors interact when health-related decisions must be made [29]. Psychological reactance is an adverse affective reaction in response to the impositions that affect the autonomy of individuals [30]. Recommendations to follow medical prescriptions have the potential to provoke reactance and, in turn, lead patients to ignore recommended treatment and adopt attitudes contrary to the message [31]. A college student sample with high reactance showed a greater threat to their freedom to a sexual health message than those low with reactance [32]. Furthermore, various studies report that psychological reactance is negatively associated with adherence to treatment and predicted risky sexual behaviors, even after controlling for other behavioral predictors [33].

Additionally, personal beliefs about medication could become barriers that limit adherence to contraceptive treatment [34]. In this sense, beliefs about the efficacy of contraceptive methods facilitate contraceptive behavior [35]. Negative beliefs about medication, such as concerns about safety and long-term effects, are associated with poor adherence in women [36]. Providing information about contraceptive methods and paying attention to patient factors promote informed decision-making and higher adherence [37]. This research has important practical implications, providing a basis for the development of adherence-enhancing interventions focusing on psychological characteristics that could promote a maintenance of behavior change in clinical practice. Moreover, if the interventions based on the social cognitive model are shown to be effective, the relevance of these psychological factors in promoting adherence would be corroborated.

In this regard, this study aimed to explain the extent these processes and attitudes participate in the prediction of non-adherence to contraception, and how they are associated with their prescription based on two medical conditions (AUP and GP). The objective was to analyze whether adherence and non-adherence to OCP is dependent on the prescription to avoid an unintended pregnancy (AUP) or gynecological problems (GP), as well as the attitudes or beliefs about medications and psychological factors (locus of control and reactance) associated differentially with it. In the case of the pill, the ‘disease’ is an unintended pregnancy, for which the level of tolerance of side effects is very low in comparison with the gynecological prescription. It was hypothesized that psychological processes, such as psychological reactance and locus of control on health, will be better predictors of adherence to contraceptive medication prescribed to an AUP group. Due to their connection with side effects, attitudinal and belief factors, however, will allow for a better prediction when contraceptive medication is prescribed to a GP group.

## 2. Materials and Methods

### 2.1. Participants

A total of 689 young women, aged 18 to 49 years (mean age = 23.41, SD = 5.91), participated in the study. Most participants had completed secondary (53.6%) and university studies (44%), while 2.4% had only attended primary school. All of them were taking oral contraception: 417 (60.52%) as a method to avoid unintended pregnancies (AUP mean age = 22.93, SD = 5.15), and 272 (39.48%) as treatment for gynecological problems (GP mean age = 24.14, SD = 7.12).

### 2.2. Instruments

Sociodemographic variables, such as age and academic background, were recorded. Moreover, information about hormonal treatment and the reasons this medication was prescribed were measured.

Attitudes and psychological processes on OCP medication were assessed based on three widely used scales: Drug Attitude Inventory–10 (DAI-10) [38], Multidimensional Health Locus of Control scale Form C (MHLC-C) [39], and The Hong Psychological Reactance Scale (HPRS) [30]. Following the procedure described in De las Cuevas and León [40], 22 items were selected (9 from DAI-10, 9 from MHLC-C, and 4 from HPRS), which showed a higher weight in each scale and greater predictive capacity of adherence. In this way, the two subscales of the DAI-10 assessed were pharmacophilia (6 items) or favorable attitude toward medication (e.g., I think the good of the medication outweighs the bad), and pharmacophobia (3 items) or negative aspects toward medication (e.g., it is unnatural for my mind and body to be controlled by drugs). The three subscales of the MHLC-C were internal health locus of control (e.g., I am directly responsible for whether my illness gets better or worse), chance health locus of control (e.g., any improvement in my health problem depends largely on luck), and doctor health locus of control (e.g., following my doctor’s instructions firmly will prevent my disease from getting worse). Finally, a global measure of psychological reactance (4 items) was obtained (e.g., when someone forces me to do something, I think about doing the opposite). Patients were asked to rate the degree to which they agree or disagree with each statement, on a 6-point Likert scale (from 1, completely disagree, to 6, completely agree). The score for each scale was obtained by adding up the scores for the constituent items. Higher scores on each subscale indicated that the characteristic was more marked. Cronbach’s alphas in this study were 0.67 for pharmacophilia, 0.40 for pharmacophobia, 0.82 for psychological reactance, 0.60 for internal health locus of control, 0.44 for doctor health locus of control, and 0.72 for chance health locus of control. The results obtained with pharmacophobia and doctor health locus of control subscales should be taken with caution, due to their low internal consistency.

The Beliefs about Medication Questionnaire (BMQ) [41] is an instrument that assesses beliefs about medical treatment. It distinguishes between perceived necessity of medication, and concern about the consequences of the treatment. It contains 10 items on a 5-point Likert scale, ranging from 1 = strongly disagree to 5 = strongly agree. The individuals with higher scores on each scale were considered to have higher necessity or concern. The Cronbach’s alpha for the validated Spanish version was 0.70 for the necessity subscale, and 0.80 for the concern subscale [39], in the same way as with the sample of this study.

The Sidorkiewicz Adherence Tool [42] assessed the level of adherence of each individual drug. The questionnaire consists of five questions: definitive default (item 1), dose failure (item 2), daily interruption (item 3), weekly default (item 4), and postponing the dose for 12 h or more (item 5). The first and second questions make up a dichotomous answer: yes = 1, no = 0, and the other three have as a possible answer: 0 = never, 1 = sometimes, 2 = frequently. In this study, item 2 was not included. The higher the scores, the higher the non-adherence to OCP. The validated Spanish version was used [43].

### 2.3. Procedure

Psychology students distributed an online link for the questionnaires through social media to females who took oral contraception. Specifically, a group of 15 psychology students were asked to encourage other women who took oral contraception to participate. The students posted the link once and kept the link active for 14 days.

Eligibility criteria for inclusion were that participants were female, in their fertile period, and had a prescribed oral contraception treatment taken daily. Exclusion criteria were not being in their fertile period, and taking hormonal treatment for serious illnesses, such as gynecological cancer.

Participants were informed about the aim of the research, and the anonymity and confidentiality of the information gathered. It was also clarified that completion of the instruments implied express consent for the data to be used for research purposes. Participants could withdraw from the study if they considered it appropriate at any time. They received no type of compensation for participating in the research.

### 2.4. Data Analysis

First, the mean differences between the two medical condition groups (AUP and GP) for the variables included in the study were analyzed through MANOVA. Second, the relationships between all the variables included in the study were analyzed (age, adherence behavior, beliefs, attitudes, and psychological factors) using Pearson’s correlation for continuous variables. Lastly, the predictors of adherence and non-adherence behavior in both medical condition groups were analyzed through logistic binary regressions.

## 3. Results

### 3.1. Descriptive and MANOVA Analysis

In both groups, OCP non-adherence was similar. Definitive default was 15.1% for AUP versus 18.4% for GP. Daily interruption of the medication was 15.1% and 15.4% in the AUP and GP groups, respectively. Weekly interruption was 33.8% for AUP and 36.4% for GP. Postponing the dose was 67.4% for AUP, and 62.1% for GP.

The MANOVA results comparing the variables of the two medical conditions (AUP and GP) of this study are shown in Table 1. There were significant differences between the two groups in five factors. The participants of the AUP group were younger, and tended to postpone the doses. Additionally, they showed higher psychological reactance, higher pharmacophobia, higher pharmacophilia, and lower doctors health locus of control than the GP group.

### 3.2. Correlational Analysis

The correlations for the psychological measures with the item of definitive default adherence are shown in Table 2 (the AUP group is placed below the diagonal, and the GP group above). Definitive default adherence was significantly associated with psychological reactance, concerns about the consequences, and pharmacophobia in the AUP group, whereas definitive default was only related to concern for the GP group. All the correlations were in the direction predicted.

### 3.3. Logistic Binary Regressions Analysis

Table 3 shows the significant psychological predictors for each item of OCP non-adherence for both medical condition groups. In the AUP group, psychological reactance was the main predictor for two OCP non-adherence items (definitive default and daily interruption). Psychological reactance explained 3.5% of the variance of default adherence. The global percentage of correctly classified cases was 84.8%, women with greater adherence being better classified (100%). Findings were similar for daily interruption: psychological reactance explained 3% of the variance, and 84.8% of total cases were correctly classified. Concern about the consequences explained 6.5% of the variance of weekly default. The global percentage of correctly classified cases was 66.7%, although the percentage of correctly classified cases was only 12.1% for the non-adherent group, and 94.5% for the adherent group. Regarding postponing the dose, the lower scores in doctor’s locus of control, and higher scores in chance locus of control and pharmacophilia were significant predictors. These variables explained 12.9% of the variance of postponing the dose. The global percentage of correctly classified cases was 67.2%, women with non-adherence being better classified (100%).

In the GP group, OCP non-adherence was less associated with psychological variables. Definitive default was only predicted by higher concerns about the consequences, which explains 3.5% of the variance. The global percentage of correctly classified cases was 81.6%, with women with greater adherence being better classified (100%). Daily interruption was explained by a higher necessity of the medication (2.9%). The global percentage of correctly classified cases was 84.6%, with women with greater adherence being better classified (100%). Pharmacophilia explained 2.6% of the variance of weekly default. The global percentage of correctly classified cases was 63.2%, although the percentage of correctly classified cases was only 6.1% for the non-adherent group, and 96% for the adherent group. Finally, postponing the dose was predicted by lower necessity of the medication and higher pharmacophilia. These variables explained 7.9% of the variance of postponing the dose. The global percentage of correctly classified cases was 63.6%, the non-adherent group (89.9%) being better classified than the adherent group (20.4%).

In general, the group that showed adherence was better classified than those who showed non-adherence. However, for postponing the dose, the non-adherence groups were better classified for both medical conditions (AUP and GP).

## 4. Discussion

The aim of this study was to identify the psychological processes and attitudes to medication involved in adherence to contraceptive pills, depending on women’s reasons for using them (unintended pregnancies or gynecological problems). The results confirmed that women’s reasons for taking contraceptive medication were related to adherence and other psychological and attitudinal variables.

In both medical conditions, medication compliance was altered in different ways. On the one hand, most women postponed the dose (over 60%), which could increase the likelihood of forgetting or interrupting medication intake. In fact, more than one third of the women forgot to take the pill at least once a week.

The AUP group was younger, postponed more doses, and showed higher pharmacophilia, pharmacophobia, and psychological reactance, and lower doctors health locus of control. Adolescent girls tended to have less adherence to oral contraceptives due to misconceptions and fear, such as thinking that the pill causes menstrual irregularities or even fertility problems [44], and preoccupations and distress [18], which may simultaneously be influencing high psychological reactance. However, the beneficial effects of OCP intake on visuospatial ability and facial affect discrimination tasks have been highlighted [45]. As hypothesized, women in the AUP group had more psychological processes involved in non-adherence than the GP group. Psychological reactance was the factor that contributed most to explaining non-adherence in women who used OCP for contraceptive purposes. Psychological reactance on many occasions implies denial of the existence of a threat [46], which could lead to a lack of rigor in taking the pill. Proposing the potential benefits of a health treatment versus the risks could decrease psychological reactance [47]. In addition, postponing the dose was associated with higher pharmacophilia, chance health locus of control, and a lower doctors health locus of control. Despite showing a favorable attitude toward OCP, possibly because of its high efficacy rates in avoiding unintended pregnancies (greater than 99% if used correctly) [48], the girls in the AUP group showed high external control based on chance, which could lead them to believe they would be lucky and not fall pregnant if they only postponed or forgot to take one of the doses. At the same time, these girls were less trusting of doctors, whose opinion they considered as an external agent that could control their behavior. People who show high doctor’s health locus of control tend to follow medical prescriptions to a greater extent, while those who show high chance health locus of control have lower adherence [49].

Women with gynecological problems reported difficulties with adherence, mainly due to their beliefs about contraceptive pills. OCP non-adherence was associated with a greater concern about the consequences, mainly related with definitive default, in line with previous studies [36]. In the same line, it has been found that women with polycystic ovary syndrome habitually expresses that beliefs about disease and the lack of information on the part of healthcare professionals greatly influence their adherence [50]. While this method is one among other contraceptive options for women who use the pill for contraceptive purposes, for women with a health problem the pill becomes a necessity. Therefore, there may be increased concern about its continued use. For gynecological problems, such as endometriosis and dysmenorrhea, it is recommended to consider whether there is a need for contraceptive medication, along with contraindications to hormonal therapy, and potential adverse effects [51]. Previous research indicated that knowledge of the risk factors of the medication is usually greater than of the beneficial effects, such as a reduced likelihood of contracting gynecological cancers, or other hormonal alterations [52,53]. In general, concerns about the consequences were the main factor involved in OCP definitive default. Concerns about side effects may have an impact on anticipatory fear of medication that could also affect regular pill-taking [54]. Additionally, lower necessity of medication and higher pharmacophilia were the more important adherence predictors for postponing the dose in the GP group. Pharmacophilia has been associated with a greater use of analgesics in older women [55]. In women with osteoporosis, findings showed that adherence to medication was related to necessity, self-efficacy, and treatment satisfaction [56]. Although women with gynecological problems showed a positive attitude toward the medication, they considered that postponing the dose would not necessarily lead to negative effects on their health. However, it would be of interest to warn these women about the danger of postponing the dose, as they may forget to take it altogether.

The results should be taken with caution, as this was a cross-sectional study with a convenience sample, which makes it impossible to obtain causal conclusions. Moreover, the sample selection procedure does not guarantee that all women were in OCP treatment at the time of responding to the self-report measures. The fact of using self-report scales may lead to a bias in the results. Nevertheless, self-reports have been shown to be effective for assessing adherence in clinical settings [43]. Some scales, such as pharmacophobia and doctor’s health locus of control, showed low internal consistency. However, support has been found for the use of these scales, and their relationship with adherence to medication [40]. Additionally, the GP group included several pathologies. Depending on the severity of the gynecological pathology, non-adherence to the OCP could have had a different impact on health. Finally, most women had a high adherence to OCP. Future research should continue to explore other psychological processes that could be involved in non-adherence.

This study has several clinical implications. First, it is especially important to pay attention to women’s age. Younger women were significantly more likely to postpone the dose than older women. It may therefore be relevant to teach strategies for remembering when and how to take the pill, and thus avoid the risks of unintended pregnancy. Moreover, it is necessary to invest in resources for providing effective reversible methods that do not require the active participation of women (implants or intrauterine devices), and allow longer-lasting contraceptive effects. Additionally, clinicians could inform women about how daily or weekly adherence affects the efficacy of OCP. Second, the reasons for non-adherence to pills were different for both groups of women. Whereas the AUP group showed higher external and psychological control, the GP group showed greater concerns about the consequences of medication, despite understanding the need for medication and having a positive attitude toward it. Clinicians can try to identify the psychological characteristics of women to know if they could be good candidates for this type of contraceptive method, based on the perceived probability of adherence. Furthermore, it may be necessary to design psychoeducational programs to adequately report the side effects of the medication, to counteract possible irrational fears about the adverse consequences of OCP. Third, more effective communication between clinicians and patients could counter some of the misconceptions about OCP, and overcome the barriers that limit adequate adherence. The interaction between patient and healthcare provider could be associated with women’s choice of contraceptive method that is consistent with the reasons or needs for initiating contraception

## 5. Conclusions

These findings indicate that attitudes toward medication and psychological processes can play an important role in adherence to OCP, and are also involved in the reasons why the pill is used. Psychological reactance was the factor that contributed most to explaining non-adherence to the OCP to prevent unintended pregnancy. Meanwhile, beliefs about the medication were associated with non-adherence when it was used for gynecological purposes. Identifying the psychological factors and beliefs linked to contraception could guide health professionals to provide counseling to women about the best contraceptive method for them, thus increasing their adherence to medication and maximizing their well-being [57,58].

## Figures and Tables

**Table 1:** Comparisons of age, beliefs, locus of control, psychological reactance, and adherence to oral contraceptives for both medical conditions.

	AUP Group (*N* = 417)		GP Group (*N* = 272)			
	*M*	*SD*	*M*	*SD*	*F*	η^2^
Age	22.94	5.16	24.14	7.12	6.55 **	0.01
Definitive default	0.15	0.36	0.18	0.39	1.22	0.00
Daily interruption	0.19	0.49	0.21	0.53	0.13	0.00
Weekly default	0.35	0.51	0.39	0.54	0.87	0.00
Postpone the dose	0.90	0.74	0.79	0.71	3.95 *	0.10
Psychological Reactance	5.18	4.32	2.96	3.46	50.71 ***	0.07
Necessity of the medication	9.84	4.27	9.35	4.55	2.08	0.00
Concern about the consequences	11.65	3.81	11.77	3.98	0.16	0.00
Pharmacophilia	15.52	6.21	12.38	5.41	46.61 ***	0.06
Pharmacophobia	6.9	3.24	6.16	3.34	8.43 **	0.01
Internal health locus of control	1.85	1.21	1.91	1.25	0.31	0.00
Chance health locus of control	3.14	1.35	2.97	1.52	2.33	0.00
Doctors health locus of control	1.78	1.05	2.10	1.13	14.50 ***	0.02

Note: AUP: avoid unintended pregnancy; GP: gynecological problems; * *p* < 0.05, ** *p* < 0.01, *** *p* < 0.001.

**Table 2:** Correlations of age, beliefs, locus of control, psychological reactance, and adherence to oral contraceptives for both medical condition groups.

	1	2	3	4	5	6	7	8	9	10
1. Age		0.11	0.05	0.04	−0.12 *	−0.02	−0.09	−0.03	0.13 *	0.11
2. Definitive default adherence	0.05		0.06	−0.02	0.13 *	0.06	0.11	−0.08	−0.05	−0.08
3. Psychological reactance	−0.08	0.14 **		0.32 ***	0.25 ***	0.37 ***	0.30 ***	0.04	0.09	0.01
4. Necessity of the medication	0.07	−0.07	0.08		0.27 ***	0.50 ***	0.11	0.04	0.00	−0.13 *
5. Concern about the consequences	−0.03	0.10 *	0.14 **	0.34 ***		0.22 ***	0.33 ***	−0.03	−0.11	−0.01
6. Pharmacophilia	−0.05	0.06	0.60 ***	0.25 ***	0.05		0.32 **	−0.01	0.10	−0.10
7. Pharmacophobia	−0.01	0.11 *	0.27 ***	−0.04	0.22 ***	0.18 ***		−0.02	−0.01	0.09
8. Internal health locus of control	0.01	−0.01	−0.09	−0.18 ***	−0.02	−0.23 ***	0.03		−0.38 ***	0.33 ***
9. Chance health locus of control	0.03	−0.02	0.13 **	0.14 **	−0.07	0.26 ***	−0.04	−0.35 ***		−0.16 **
10. Doctors health locus of control	−0.07	−0.02	−0.18 ***	−0.08	0.09	−0.27 ***	−0.08	0.33 ***	−0.16 **	

Note: Avoid unintended pregnancy group (AUP group) is shown below the diagonal; gynecological problems group (GP group) is shown above it; * *p* < 0.05, ** *p* < 0.01, *** *p* < 0.001.

**Table 3:** Predictors of non-adherence versus adherence to oral contraceptives for both medical conditions.

	AUP Group						GP Group				
				95% Exp (B)						95% Exp (B)	
Variables	β	Wald	Exp (B)	Lower	Upper	Variables	β	Wald	Exp (B)	Lower	Upper
*Default adherence*			0.18			*Default adherence*			0.22		
Constant Psychological reactance	0.09	8.51 **	1.09	1.03	1.16	Constant Concern about the consequences	0.09	5.84 *	1.10	1.02	1.18
*Daily interruption*			0.18			*Daily interruption*			0.18		
Constant Psychological reactance	0.08	7.31 **	1.09	1.02	1.15	Constant Neccesity for the medication	0.07	4.80 *	1.08	1.01	1.15
*Weekly default*			0.51			*Weekly default*			0.57		
Constant Concern about the consequences	0.12	18.88 ***	1.13	1.07	1.20	Constant Pharmacophilia	0.05	5.23 *	1.05	1.01	1.10
*Postponing the dose*			2.05			*Postponing the dose*			1.64		
Constant Pharmacophilia	0.07	13.49 ***	1.08	1.03	1.12	Constant Neccesity for the medication	−0.09	8.49 **	0.91	0.85	0.97
Chance health locus of control	0.18	5.07 *	1.20	1.02	1.41	Pharmacophilia	0.11	13.21 ***	1.12	1.05	1.19
Doctors health locus of control	−0.26	5.83 *	0.77	0.63	0.95						

Note: AUP: avoid unintended pregnancy; GP: gynecological problems; * *p* < 0.05, ** *p* < 0.01, *** *p* < 0.001.

## Data Availability

The data presented in this study are available on request from the corresponding author.
